# Neighborhood disorder and screen time among 10-16 year old Canadian youth: A cross-sectional study

**DOI:** 10.1186/1479-5868-9-66

**Published:** 2012-05-31

**Authors:** Valerie Carson, Ian Janssen

**Affiliations:** 1 School of Kinesiology and Health Studies, Queen’s University, 28 Division St., Kingston, ON, K7L 3N6, Canada; 2 Department of Community Health and Epidemiology, Queen’s University, Kingston, ON, Canada

**Keywords:** Screen time, Neighborhood disorder, Youth

## Abstract

**Background:**

Screen time activities (e.g., television, computers, video games) have been linked to several negative health outcomes among young people. In order to develop evidence-based interventions to reduce screen time, the factors that influence the behavior need to be better understood. High neighborhood disorder, which may encourage young people to stay indoors where screen time activities are readily available, is one potential factor to consider.

**Methods:**

Results are based on 15,917 youth in grades 6-10 (aged 10-16 years old) who participated in the Canadian 2009/10 Health Behaviour in School-aged Children Survey (HBSC). Total hours per week of television, video games, and computer use were reported by the participating students in the HBSC student questionnaire. Ten items of neighborhood disorder including safety, neighbors taking advantage, drugs/drinking in public, ethnic tensions, gangs, crime, conditions of buildings/grounds, abandoned buildings, litter, and graffiti were measured using the HBSC student questionnaire, the HBSC administrator questionnaire, and Geographic Information Systems. Based upon these 10 items, social and physical neighborhood disorder variables were derived using principal component analysis. Multivariate multilevel logistic regression analyses were used to examine the relationship between social and physical neighborhood disorder and individual screen time variables.

**Results:**

High (top quartile) social neighborhood disorder was associated with approximately 35-45% increased risk of high (top quartile) television, computer, and video game use. Physical neighborhood disorder was not associated with screen time activities after adjusting for social neighborhood disorder. However, high social and physical neighborhood disorder combined was associated with approximately 40-60% increased likelihood of high television, computer, and video game use.

**Conclusion:**

High neighborhood disorder is one environmental factor that may be important to consider for future public health interventions and strategies aiming to reduce screen time among youth.

## Background

Sedentary behavior refers to sitting and lying activities that involve minimal body movement and low energy expenditure [1,2]. Sedentary behavior should not be confused with a lack of moderate-to vigorous-intensity physical activity (MVPA). These two behaviors are poorly correlated [3] and independently predict health outcomes [4,5]. A substantial amount of young peoples’ time, approximately 8.5 hours per day or 60% of all time spent awake, is devoted to sedentary behavior [6]. A large portion of this sedentary behavior time (>4.5 hours/day) is comprised of screen time activities such as watching television, using a computer, and playing video games [7]. Screen time activities have been linked to several negative health outcomes such as increased violence, low quality of life, poor body self-image, engagement in risky behaviors (e.g., drug and alcohol use), and cardiometabolic risk factors (e.g., obesity, high blood pressure) [4,5,8-12]. Thus, reducing screen time could be an important component of intervention strategies aiming to improve the health of young people.

Behavior change theories stipulate that to effectively change a behavior, such as reducing screen time, the factors that influence the behavior need to be understood in order to develop evidence-based interventions [13,14]. Two recent narrative literature reviews identified several demographic (e.g., sex, ethnicity, parents’ education/income) and social (e.g., parents’ viewing habits, number of parents in the home) factors that may influence screen time [15] and television use [16] among children and youth. However, both reviews reported that there is insufficient evidence to draw conclusions on many of the other potential correlates, especially for non-television types of screen time [15,16]. Of particular note was the lack of previous research examining the relationship between the neighborhood environment and screen time behaviors.

Ecological models suggest that the neighborhood environment is an important component in understanding health behaviors [17]. For example, the basic premise of the ecological systems theory is that a young person’s neighborhood environment interacts with their immediate context (i.e., family, peers) and their individual characteristics (i.e., age, gender) to influence their behaviors [18]. Neighborhood disorder is one aspect of the neighborhood environment that may influence sedentary behaviors such as screen time. Neighborhood disorder encompasses many factors such as crime, graffiti, abandoned buildings, gang activity, drug sales, and prostitution [19,20]. It has been suggested that the fear and distress associated with high neighborhood disorder may result in people avoiding or minimizing their time in the neighborhood environment [21]. Therefore, high neighborhood disorder may encourage young people to stay indoors where screen time activities are readily available.

Existing research on the relationship between aspects of neighborhood disorder and screen time among young people has been inconsistent. Four studies have reported little or no association between screen time use with certain dimensions of neighborhood disorder assessed by parental perceptions, such as crime [22,23] and safety [7,24]. Conversely, two studies have reported that children are more likely to be high screen time users if they live in neighborhoods with high crime rates [25] or if parental perceptions of neighborhood safety is low [26]. Additionally, physical disorder (e.g., litter, graffiti) has been linked to an increase in television viewing [27].

Limitations within the existing literature may explain the inconsistent results. For example, no study has comprehensively examined the relationship between neighborhood disorder and screen time behaviors using a variety of objective and subjective (i.e., perceptions) measures. Furthermore, many of the studies have considered the impact of certain dimensions of neighborhood disorder on a summary screen time measure (e.g., television + computer + video games) [7,23,24] or a television only measure [10,26]; however, the correlates of television, computer, and video game use may differ [28]. Thus, future research that addresses these limitations may assist in the understanding of this relationship.

Therefore, the purpose of this study was to comprehensively examine the relationship between neighborhood disorder with television, computer, and video game use. We had the opportunity to study these relationships in a large and representative sample of Canadian youth and measured neighborhood disorder using several objective and subjective items. It is anticipated that the findings of this study will provide valuable information regarding the impact of the neighborhood environment on individual screen time activities and will inform the development of evidence-based interventions aimed at reducing screen time among youth.

## Methods

### Participants

The study is based on the 2009/10 *Health Behaviour in School-aged Children Survey* (HBSC) conducted in Canada. The HBSC is a World Health Organization sponsored cross-sectional survey conducted in 44 countries. The 2009/10 Canadian HBSC is comprised of three main components: 1) a questionnaire completed by classes of students that asks about their health behaviors, lifestyle factors, and demographics, 2) a questionnaire completed by an administrator (e.g., principal) of the students’ school that asks about school policies and features of the school and its surrounding neighborhood [29], and 3) geographic information systems (GIS) measures of features in the school neighborhoods.

The Canadian sample of students was designed according to the international HBSC protocol in that a cluster design was used with school class being the basic cluster. Youth in private and special needs schools, street youth, and the incarcerated were excluded. Since this includes <10% of students in Canada the distribution reflected the distribution of Canadians in grades 6-10 (aged 10-16 years old). The sample was weighted to account for oversampling of certain provinces and territories. The total sample consisted of 26,078 students who attended 436 different schools from across Canada. A total of 36 schools (1513 students) were excluded because they did not return the administrator questionnaire. Additionally, 43 schools (2394 students) were excluded because of incomplete information on exposure variables within the administrator questionnaire. Finally, 75 schools (3419 students) were excluded because they did not have adequate Google Earth Street View information (as explained below). These excluded schools were located in rural areas or in the remote Northern Territories of Canada. We further excluded 2835 students with incomplete information on outcome and/or covariate variables, leaving 15,917 who attended 291 schools. There were no significant differences in body mass index (*P* > 0.05), between the participants that were included or excluded from the final sample. However, the final sample was slightly older (0.2 years) and included more females (4.6%), Caucasian participants (8.0%), participants of high socioeconomic status (14.0%), and large urban participants (71.5%; *P* < 0.01). Ethics approval was obtained from the Queen’s University General Research Ethics Board. Consent was also obtained from participating school boards, individual schools, parents, and students.

### Neighborhood disorder (exposure)

Ten items of neighborhood disorder were measured using the HBSC student questionnaire (2 items), the HBSC administrator questionnaire (4 items), and GIS (4 items). Consistent with previous research examining neighborhood disorder, these 10 items were classified into social (6 items) and physical (4 items) neighborhood disorder categories [19,20,30].

#### HBSC student questionnaire

Two neighborhood disorder items were included in the student questionnaire that asked the students their perception of the area where they live. Both of these were classified as social disorder (*“it is safe for younger children to play outside during the day”* and *“most people around here would try to take advantage of you if they got the chance”*). Both items had a 5-point response scales ranging from “*1 = strongly agree”* to *“5 = strongly disagree”.* After reverse-coding the second item, average values for students at each school were calculated and each student from that school was assigned that value. Thus, although these items were assessed at the individual-level, for statistical analyses and conceptual purposes they were treated as area-level variables.

#### HBSC administrator questionnaire

Four items from the HBSC administrator questionnaire inquired about the safety of the neighborhood where the school was located. All four items were classified as social disorder and assessed racial/ethnic/religious tensions, drugs/drinking in public, gangs, and crime*.* Each item had a 4-point response scale ranging from “*1 = not a problem”* to *“4 = major problem.”* Student participants were assigned their school’s score for each item.

#### Geographic information systems (GIS) and Google earth street view

Four neighborhood disorder items were measured with GIS using the CanMaps Streetfiles (DMTI Spatial Inc., Markham, ON) in ArcView version 9.3 software (ESRI, Redlands, CA), and Google Earth Street View. All four items were classified as physical disorder (litter, graffiti, vacant or abandoned buildings, conditions of buildings/grounds). In GIS, 15 observation points within a 1 km circular buffer surrounding each school were systematically plotted. The first point was at the schools’ address and the remaining 14 points were evenly spaced (approximately 500 m apart in the x and y direction) using a grid pattern. Previous research using the HBSC survey has shown that 1 km buffer measures for other social constructs (e.g., neighborhood SES) are highly correlated and related similarly to health outcomes [31]. The same 1 km circular buffer and grid pattern were used for all schools so there was consistency in the location of observation points being examined for all schools. Some variation did exist when an observation point in the grid pattern did not fall directly on a road; however, in this situation the point was relocated to the nearest road within GIS. A total of 6540 observation points were plotted across Canada and exported to Google Earth for assessment.

At each of the 6540 observation points, a single trained rater did a 360° panoramic view in Google Earth Street View to assess each of the 4 items (litter, graffiti, vacant or abandoned buildings, and conditions of buildings/grounds). When Google Earth Street View was unavailable for a single point, the point was moved within Google Earth to the nearest road within the 1 km radius. A similar procedure was followed if there were less than 3 buildings in the 360° view area for a single point. Schools were excluded if Google Earth Street View was unavailable for all or the majority of roads within the entire 1-km radius, leaving 5415 observation points.

The criteria used to assess the 4 items (litter, graffiti, vacant or abandoned buildings, and conditions of buildings/grounds) were based upon previous neighborhood disorder studies that relied on in-person assessments of the neighborhoods or videotaped assessments of the neighborhoods made from vehicle-mounted cameras travelling through the neighborhoods [20,32,33]. For the present study, litter was assessed on a 5-point scale: “*none (no pieces)*”, “*very little (1-2 pieces)*”, “*a little (3-10 pieces)*”, “*moderate amount (11-20 pieces)*”, and “*considerable amount (more than 20 pieces)*” [33]. Graffiti was assessed on a 4-point scale: “*none (no tags)*”, “*a little (1-2 tags)*”, “*moderate amount (3-5 tags)*”, and “*considerable amount (more than 5 tags)*” [33]. Numbers of vacant or abandoned buildings were assessed on a 4-point scale: “*none (no buildings are vacant)*”, “*less than one third (if 10 buildings, 1-2 are vacant)*”, “*one third to one half (if 10 buildings, 3 to 5 are vacant)*”, and “*more than half (if 10 buildings, more than 5 are vacant)*” [33]. Overall condition of the buildings were assessed on a 4-point scale: “*excellent*”, “*good*”, “*fair*” and “*poor*” and was based on both building (walls, windows, stairs, roof) and ground (landscaping, lawn, driveway) characteristics [33]. School averages for all 4 items were calculated based on the ratings of the 15 observation points.

Reliability assessments for the 4 items (litter, graffiti, vacant or abandoned buildings, and conditions of buildings/grounds) were calculated based on 150 observation points around 10 randomly selected schools. The intra-rater reliability, performed by the original rater (who assessed the 6540 observation points for the 4 items) one week apart, was r = 0.94 for litter, r = 0.99 for graffiti, r = 0.78 for vacant or abandoned buildings, and r = 0.82 for conditions of buildings/grounds. The inter-rater reliability (performed by original rater and a second independent rater) was r = 0.90 for litter, r = 0.95 for graffiti, r = 0.80 for vacant or abandoned buildings, and r = 0.75 for conditions of buildings/grounds.

Validity of the 4 items was determined by comparing Google Earth Street View assessments and in-person assessments throughout the city of Kingston, Ontario. The original rater was also used in the validity sub-study assessments; however, they were blinded to the Street View assessments when completing the in-person assessments. A total of 521 points were evenly spread (approximately 500 m apart in the x and y direction) across the entire city of Kingston using a grid pattern. The validity was r = 0.65 for litter, r = 0.76, for graffiti, r = 0.99 for vacant or abandoned buildings, and r = 0.91 for conditions of buildings/grounds. Note that for the validity assessment there was an approximate 2 year time difference between when the Google Earth Street View images were obtained (summer of 2009) and when the in-person assessments were made (summer of 2011). Our validity coefficients are higher than previous studies that have assessed the validity of Google Earth Street View for measuring neighborhood environment features [34,35]. However, these studies tended to focus on more detailed features (i.e., broken glass) [34] and had larger time-gaps between assessments [34]. Furthermore, they did not rigorously train the individuals who obtained the measures [35].

#### Creation of neighborhood disorder scores

Principal component analysis was conducted in order to reduce the six social and four physical neighborhood disorder items. For social disorder, one component with an eigenvalue of 3.4 emerged, explaining 55.8% of the total variance. The items that were included in this component and their loadings were 0.56 for neighbours taking advantage, 0.58 for safety, 0.77 for using drugs/drinking in public, 0.78 for racial/ethnic/religious tensions, 0.86 for crime, and 0.87 for gangs. For physical disorder, one component with an eigenvalue of 2.5 emerged, explaining 62.1% of the total variance. The items that were included in this component and their loadings were, 0.74 for conditions of buildings/grounds, 0.78 for vacant or abandoned buildings, 0.81 for litter, and 0.83 for graffiti. The Anderson-Rubin method was used to calculate z-scores for the components that were derived from the social and physical neighborhood disorder principal components analyses. Therefore, the scores had a mean of zero and a standard deviation of one [36].

### Screen time (outcome)

The amount of hours spent watching television, playing video games, and using the computer per weekday and weekend were determined using 6 questions [29]. For each question there was a 9-point response scale ranging from “*none at all*” to “*7 or more hours a day*”. Weighted means for weekday and weekend use were used to calculate the total hours per week (average weekday screen time*5 + average weekend screen time*2). Each screen time variable was categorized into quartiles and the highest quartile was used to denote high use. A previous validation study examined a brief questionnaire used to measure television viewing time, similar to that used in HBSC. A significant correlation (r = 0.47) was observed with television viewing time measured by a weekly log among 11- to 15-year-olds [37].

### Covariates

Covariates included gender, grade, ethnicity (Caucasian, other), family structure, individual-level socioeconomic status (SES), area-level SES, and urban-rural location. Family structure was measured by asking participants who they lived with all or most of the time and the following groups were created: both parents, single parent, parent and step parent, and other [38]. Individual-level SES was measured using the previously validated family affluence scale, which includes the summation of 4 items regarding family wealth (car ownership, bedroom sharing, holiday travel, and computer ownership) [39]. Participants were divided into low, medium, and high groups based on previously established cut-points [39]. Area-level SES was calculated using 2006 Canadian census data in PCensus for MapPoint through GIS. Three measures of area-level SES were obtained for the census subdivisions in the 1 km radius around participating schools including: education (percentage of adult residents with less than a high school education), income (average employment income), and unemployment rate. Principal component analysis was used to create a continuous summary score based on the inverse of education, the inverse of unemployment rate, and income. Urban-rural location was based on the population of the municipality where the school was located. This was calculated using 2006 Canadian census data in PCensus for MapPoint through GIS. Schools were classified as either rural (population < 1,000), small urban (population of 1,000-10,000), or large urban (population >10,000) [40].

### Statistical analysis

Analyses were completed using SAS version 9.2 (SAS Institute Inc., Cary, NC). Descriptive statistics were initially calculated, including average weekly hours of television, video game, and computer use per social and physical neighborhood disorder quartiles. Additionally, t-tests were used to explore gender differences in individual screen time activities. Multivariate multilevel logistic regression analyses were used to examine the relationship between social and physical neighborhood disorder and individual screen time variables. The GLIMMIX procedure was used to fit generalized linear mixed models with a binomial distribution to account for the sample weights as well as the hierarchical and clustered nature of the data. All models predicted the highest quartile of television, computer, and video game use.

To address the main objective of the paper, initial unadjusted regression models were run for each screen time variable that included social or physical neighborhood disorder. Then, a second set of models were run that included social or physical neighborhood disorder variables and relevant confounders. Potential confounders for the models included gender, grade, ethnicity, family structure, individual-level SES, area-level SES, and urban-rural location. These potential confounders were based on assumptions of confounding [41] as well as previous literature on the relationship between the neighborhood environment and screen time [5,23,25,26]. A two-stage backward deletion procedure was used to select the confounders [41]. Confounders were removed based on a change of less than 10% on the main effects [41]. In stage one, an initial model was run to select individual-level confounders. In stage two, the selected individual-level confounders were then added to an additional model with area-level confounders for a final selection process. A third set of models were run that included the relevant confounders and both the social and physical neighborhood disorder variables. Gender, grade, ethnicity, and SES interactions were also explored. Finally, since the screen time categories were not rare outcomes, odds ratios (ORs) obtained from logistic regression models do not approximate relative risk. Therefore, prevalence ratios (PRs) were derived by adjusting ORs according to the proportion of the outcome in the referent groups (P_0_) as follows: PR = OR/((1-P_0_) + (P_0_*OR)) [42].

While some participants did not live within 1 km of their school where the neighborhood disorder was measured, these participants traveled through the 1 km buffer to and from school. Furthermore, students tend to spend a considerable amount of time in and around their schools before and after the school day. Nevertheless, sensitivity analyses were conducted to examine whether the results for the total sample were consistent with the sample of participants who lived within 1 km of their school. All the aforementioned analyses were re-run in the subsample of participants who, based on their available residential postal code, were calculated to live within the 1 km circular buffer of their school. This distance was calculated by placing a point on the geographic center of the postal codes, which were typically street blocks. Approximately 66% of the final sample from 248 schools provided their residential postal codes. Approximately 40% of these participants lived within 1 km of their school, approximately 60% lived within 2 km, approximately 70% lived within 3 km, and approximately 80% lived within 5 km.

We also estimated the combined influence of social and physical neighborhood disorder on screen time by dividing participants into 4 groups based on the quartile values of social and physical neighborhood disorder. The four groups included, 1) low social and physical neighborhood disorder (low social/low physical), 2) high social neighborhood disorder and low physical neighborhood disorder (high social/low physical), 3) low social neighborhood disorder and high physical neighborhood disorder (low social/high physical), and 4) high social and physical neighborhood disorder (high social/high physical). Low represents the bottom 3 quartiles and high represents the top quartile. Multivariate multilevel analyses were conducted to predict high television, computer, and video game use.

Additive and multiplicative interactions were assessed by comparing the observed and expected joint effects. An interaction was considered present if the observed and expected effect were substantially different [43]. The observed effect for the high social/high physical group was the estimated prevalence ratio. For additive interactions, the expected effect for the high social/high physical group was calculated by summing the estimated prevalence ratios for the high social/low physical group and the low social/high physical group and then subtracting by 1 [43]. For multiplicative interactions, the expected effect for the high social/high physical group was calculated by multiplying the estimated prevalence ratios for the high social/low physical group and the low social/high physical group [43]. A test of homogeneity of stratified estimates was also calculated to determine whether there were statistically significant differences between observed and expected effects [43].

## Results

Participant characteristics are shown in Table 1. For the total sample (N = 15,917), approximately 52% were female and the average age was 14 (1.5 SD) years. The average weekly hours of television, computer, and video game use were 17.5, 14.5, and 12.8, respectively (Table 1). There were significant gender differences in screen time. More specifically, boys engaged in more television (1.3 hrs/wk) and video game (8.4 hrs/wk) use; whereas, girls engaged in more computer (2.8 hrs/wk) use. The participant characteristics of the sensitivity analyses subsample (N = 4,163) were similar to those of the total sample (Table 1). Average weekly hours of television, computer, and video games increased (P_trend_ < 0.01) across social neighborhood disorder and physical neighborhood disorder quartiles (Table 2).

**Table 1:** Characteristics of the total sample (N = 15,917) and participants living within 1 km of their school (N = 4,163)

	***Total (N = 15,917)***	***Sensitivity Analyses Subsample (N = 4,163)***
Sex (%)		
Male	47.6	47.7
Female	52.4	52.3
Grade (%)		
6	17.7	25.3
7	18.9	21.1
8	21.2	22.6
9	21.2	16.4
10	21.0	14.5
Ethnicity (%)		
Caucasian	76.7	73.1
Other	23.3	26.9
Family Structure (%)		
Both Parents	61.6	63.1
Single Parent	17.0	16.8
Parent and Step Parent	9.8	9.5
Other	11.6	10.6
Individual-level Socioeconomic Status (%)		
Low	7.6	7.3
Medium	35.5	35.6
High	56.9	57.1
Urban-rural Location (%)		
Rural	4.0	8.4
Small Urban	23.7	31.2
Large Urban	72.3	60.4
Screen Time, hours/week		
Television	17.5 (12.1)	17.9 (12.0)
Computer	14.5 (13.1)	14.0 (12.7)
Video Games	12.8 (13.3)	13.1 (12.8)

Data presented as prevalence for the categorical variables and as mean (standard deviation) for the continuous variables.

**Table 2:** Mean weekly hours of screen time within social and physical neighborhood disorder quartiles (N = 15,917)

**Variable**	**Television**	**Computer**	**Video Games**
Social Neighborhood Disorder			
Quartile 1	16.2 (11.4)	12.8 (12.2)	11.9 (12.7)
Quartile 2	17.2 (11.9)	14.4 (12.6)	12.1 (12.8)
Quartile 3	17.4 (11.9)	14.2 (12.8)	12.6 (13.1)
Quartile 4	19.3 (13.1)	16.3 (14.2)	14.4 (14.2)
	P _trend_ < 0.01	P _trend_ < 0.01	P _trend_ < 0.01
Physical Neighborhood Disorder			
Quartile 1	16.6 (11.6)	13.7 (12.5)	12.1 (12.5)
Quartile 2	17.7 (12.2)	14.6 (13.3)	12.7 (13.3)
Quartile 3	16.8 (11.7)	14.2 (12.8)	12.6 (13.3)
Quartile 4	19.2 (12.8)	15.3 (13.5)	13.8 (13.8)
	P _trend_ < 0.01	P _trend_ < 0.01	P _trend_ < 0.01

Data presented as mean (standard deviation).

The associations between neighborhood disorder and screen time in the total sample (N = 15,917) are shown in Table 3. The prevalence of participants who were high (top quartile) television, computer, and video game users increased across social neighborhood disorder and physical neighborhood disorder quartiles (P_trend_ < 0.01). The multilevel regression analyses, adjusted for confounders (model 2) and physical neighborhood disorder (model 3), indicated that social neighborhood disorder was independently related to high television, computer, and video game use. By comparison to youth in quartile 1, the prevalence ratios for youth in quartile 4 were 1.33 (95% confidence interval: 1.16-1.54) for high television use, 1.46 (1.30-1.65) for high computer use, and 1.42 (1.25-1.59) for video games use. The multivariate analyses (model 3 in Table 3) for the physical neighborhood disorder measure indicated that physical neighborhood was not an independent predictor of high television, computer, or video game use. No meaningful gender, grade, ethnicity, and SES interaction effects were observed.

**Table 3:** Multi-level models predicting screen time use in the total sample (N = 15,917)

	**Prevalence%**	**Model 1**		**Model 2**		**Model 3**	
		**PR**	**95% CI**	**PR**	**95% CI**	**PR**	**95% CI**
*Television*							
Social Neighborhood Disorder							
Quartile 1	21.6	1.00		1.00		1.00	
Quartile 2	21.7	1.03	0.86 - 1.21	1.02	0.86 - 1.20	1.02	0.85 – 1.19
Quartile 3	26.6	1.28	1.12 – 1.48*	1.22	1.05 – 1.42*	1.19	1.02 – 1.38*
Quartile 4	32.1	1.45	1.26 – 1.65*	1.39	1.20 – 1.58*	1.33	1.16 – 1.54*
Physical Neighborhood Disorder							
Quartile 1	22.5	1.00		1.00		1.00	
Quartile 2	25.4	1.13	0.96 – 1.32	1.05	0.88 – 1.23	1.01	0.85 – 1.18
Quartile 3	23.7	1.06	0.91 – 1.26	0.98	0.80 – 1.17	0.92	0.76 – 1.11
Quartile 4	30.6	1.43	1.23 – 1.63*	1.28	1.08 – 1.49*	1.17	0.98 – 1.37
*Computer*							
Social Neighborhood Disorder							
Quartile 1	21.2	1.00		1.00		1.00	
Quartile 2	25.8	1.17	1.01 – 1.36*	1.07	0.92 – 1.23	1.05	0.91 – 1.22
Quartile 3	27.8	1.34	1.16 – 1.52*	1.15	1.00 – 1.32*	1.13	0.98 – 1.29
Quartile 4	34.8	1.66	1.51 – 1.90*	1.50	1.34 – 1.68*	1.46	1.30 – 1.65*
Physical Neighborhood Disorder							
Quartile 1	24.4	1.00		1.00		1.00	
Quartile 2	27.7	1.20	1.03 – 1.41*	1.18	1.00 – 1.39*	1.11	0.95 – 1.28
Quartile 3	26.9	1.14	0.96 – 1.36	1.12	0.93 – 1.34	1.02	0.85 – 1.20
Quartile 4	30.4	1.36	1.18 – 1.57*	1.32	1.09 – 1.55*	1.14	0.97 – 1.33
*Video Games*							
Social Neighborhood Disorder							
Quartile 1	20.7	1.00		1.00		1.00	
Quartile 2	22.8	1.14	1.00 – 1.31*	1.14	1.00 – 1.31*	1.14	0.99 – 1.31
Quartile 3	25.5	1.24	1.09 – 1.40*	1.20	1.05 – 1.35*	1.18	1.03 – 1.33*
Quartile 4	30.6	1.48	1.31 – 1.65*	1.44	1.28 – 1.60*	1.42	1.25 – 1.59*
Physical Neighborhood Disorder							
Quartile 1	22.7	1.00		1.00		1.00	
Quartile 2	24.2	1.08	0.94 – 1.24	1.03	0.89 – 1.19	1.03	0.90 – 1.18
Quartile 3	25.1	1.11	0.95 –1.28	1.05	0.89 – 1.22	1.03	0.89 – 1.19
Quartile 4	27.6	1.26	1.10 – 1.43*	1.17	1.01 – 1.34*	1.13	0.98 – 1.28

PR = prevalence ratio; 95% CI = 95% confidence interval; * *P* ≤ 0.05.

Model 1: unadjusted. Model 2 adjusted for neighborhood SES and model 2 of social neighborhood disorder predicting high computer use was also adjusted for grade. Model 3 adjusted for confounders in model 2 and physical neighborhood disorder or social neighborhood disorder.

The multivariate analyses mentioned in the preceding paragraph were repeated for the sample of participants (N = 4,163) who lived within 1 km of their school (Table 4). The same patterns of observations were observed. Furthermore, the prevalence ratios observed for the subsample in Table 4 were of a similar order of magnitude to those observed for the entire sample in Table 3.

**Table 4:** Multi-level models predicting high screen time use for participants living within 1 km of their school (N = 4,163)

	**Television**		**Computer**		**Video Games**	
	**PR**	**95% CI**	**PR**	**95% CI**	**PR**	**95% CI**
Social Neighborhood Disorder						
Quartile 1	1.00		1.00		1.00	
Quartile 2	0.96	0.74 – 1.24	1.05	0.82 – 1.35	1.02	0.79 – 1.27
Quartile 3	1.01	0.77 – 1.29	1.01	0.77 – 1.29	0.98	0.77 – 1.23
Quartile 4	1.32	1.05 – 1.62*	1.27	1.00 – 1.57*	1.30	1.07 – 1.58*
Physical Neighborhood Disorder						
Quartile 1	1.00		1.00		1.00	
Quartile 2	0.93	0.70 – 1.20	0.88	0.67 – 1.14	1.02	0.80 – 1.28
Quartile 3	1.01	0.76 – 1.31	1.04	0.79 – 1.33	1.13	0.86 – 1.41
Quartile 4	1.11	0.83 – 1.45	1.12	0.86 – 1.45	1.05	0.80 – 1.33

PR = prevalence ratio; 95% CI = 95% confidence interval; * *P* ≤ 0.05.

All models were adjusted for neighborhood SES. The social neighborhood disorder model predicting high computer use was also adjusted for grade. All social neighborhood disorder models were also adjusted for physical neighborhood disorder. All physical neighborhood disorder models were also adjusted for social neighborhood disorder.

**Figure 1:**
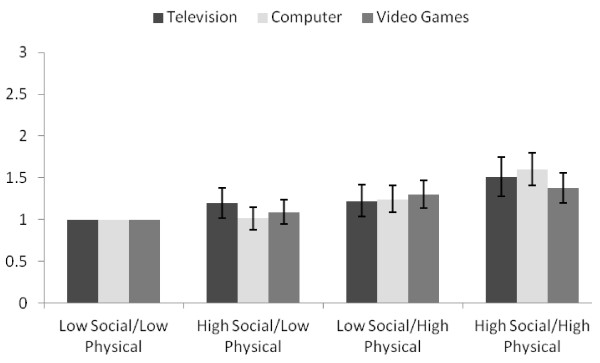
**The combined influence of social and physical neighborhood disorder on television, computer, and video games.** Prevalence ratios and 95% confidence intervals for high television, computer, and video game use according to combined social and physical neighborhood disorder groups. All models were adjusted for neighborhood SES and the computer model was also adjusted for grade. Low social/low physical = bottom 3 quartiles of social and physical neighborhood disorder; high social/low physical = top quartile of social neighborhood disorder and bottom 3 quartiles of physical neighborhood disorder; low social/high physical = bottom 3 quartiles of social neighborhood disorder and top quartile of physical neighborhood disorder; and high social/high physical = top quartiles of social and physical neighborhood disorder.

The relationship between combined social and physical neighborhood disorder and screen time are shown in Figure 1. Based upon the prevalence ratios for high television use observed in the high social/low physical group (1.20, 1.02-1.38) and the low social/high physical group (1.22, 1.04-1.42), the observed prevalence ratio in the high social/high physical group (1.51, 1.28–1.75) was greater than that expected from both the additive (1.42) and multiplicative (1.46) interaction assessment. Similarly, based upon the prevalence ratios for high computer use observed in the high social/low physical group (1.02, 0.88-1.15) and the low social/high physical group (1.24, 1.09-1.41), the observed prevalence ratio in the high social/high physical group (1.60, 1.41–1.80) was greater than that expected from both the additive (1.26) and multiplicative (1.27) interaction assessment. Conversely, based upon the prevalence ratios for high video game use observed in the high social/low physical group (1.09, 0.95–1.24) and the low social/high physical group (1.30, 1.14–1.47), the observed prevalence ratio in the high social/high physical group (1.38, 1.20–1.56) was less than that expected from both the additive (1.39) and multiplicative (1.42) interaction assessment. However, further evaluation using the test of homogeneity of stratified estimates suggested that social and physical disorder interactions were not present for any of the screen time variables (*P* > 0.05).

## Discussion

This study examined the effects of social and physical neighborhood disorder on television, computer, and video game use in a large cross-sectional sample of Canadian youth. High social neighborhood disorder was associated with a 35-45% increased risk of high television, computer, and video game use. Physical neighborhood disorder was not associated with screen time activities after adjusting for social neighborhood disorder. However, high social and physical neighborhood disorder combined was associated with approximately 40-60% increased risk of high television, computer, and video game use.

To our knowledge, seven previous studies have examined the influence of certain aspects of social and physical neighborhood disorder on screen time among youth. The findings of these studies are mixed. In terms of social neighborhood disorder, four studies reported that parental perceptions of crime [22,23] and safety [7,24] had little or no influence on screen time use among school-aged children and adolescents. Whereas, two studies reported modest overall associations between high crime rates [25] and low parental perceptions of neighborhood safety [26] with screen time. Additionally, one study reported a link between physical disorder and television viewing among 5-year-olds [27] .

The inconsistency in findings across the previous literature may be due to the measure of neighborhood disorder used. For example, six out of the seven previous studies only considered one dimension of neighborhood disorder such as safety or crime [27]. Thus, these studies may not have fully conceptualized the measure of neighborhood disorder [30]. In addition, all seven previous studies used either a subjective measure (i.e., parent perceptions) or an objective measure (i.e., crime statistics) of neighborhood disorder. However, several investigators have suggested that in order to make a comprehensive assessment of the environment’s condition and accurate inferences regarding the environment’s effect, both objective measures and perceptions of the environment should be considered [30,33,44-47], as was done in our study.

The prevalence ratios for screen time observed in the highest social and physical neighborhood disorder quartiles in our study were in the order of 1.13 to 1.60, which would be considered weak to modest effect sizes by epidemiological standards [48]. With that being said, it is important to recognize that exposures measured at the area-level, such as neighborhood disorder in the present study, tend to have smaller effect sizes than individual-level exposures [49]. When compared to the influence that other area-level exposures have on screen time use (e.g., urban-rural status [50] or neighborhood SES [51,52]) the risk estimates for social neighborhood disorder observed here and of previous studies [7,25] were of a similar order of magnitude.

Our findings are supported by ecological models, which recognize the importance of multiple levels of influence, including the neighborhood environment, on health behaviors [17]. Furthermore, our findings are supported by the neighborhood disorder model [21]. High neighborhood disorder has consistently been linked with psychological distress [53]. The premise of the neighborhood disorder model is that high neighborhood disorder negatively influences mental health partly through fear [21]. It has been suggested that people may cope with this fear and distress by minimizing or avoiding their exposure to their neighborhood environment [21]. Thus, youth in the present study that lived in neighborhoods with high neighborhood disorder may have been more inclined to stay indoors to avoid dangerous situations and other deviant behavior. When young people are indoors they are more likely to engage in screen time activities because they are highly accessible [54].

The present study suggests that social neighborhood disorder is more strongly associated with screen time than physical neighborhood disorder. It is important to note that social neighborhood disorder was assessed through subjective measures (i.e., perceptions), while physical neighborhood disorder was assessed through objective measures. Therefore, differences in findings could be due to differences in measures. However, Molnar and colleagues, who assessed both social and physical neighborhood disorder through objective measures, also reported that social neighborhood disorder was more strongly associated with youths’ recreational physical activity than physical neighborhood disorder [20]. Combined, these observations suggest that high social neighborhood disorder may have a greater influence on whether youth stay indoors, compared to high physical neighborhood disorder. However, the greatest influence on screen time use was observed when examining the combined effects of the variables. Participants living in neighborhoods with both high social and physical neighborhood disorders were approximately 40-60% more likely to be high television, computer, and video game users compared to participants in neighborhoods of low social and physical disorder.

The majority of screen time reduction interventions conducted thus far have been individual or family-focused [55]. However, according to a recent systematic review, these interventions have been largely ineffective in reducing screen time [55]. While there were several methodological concerns with the available evidence in the systematic review [55], future research is still needed to better understand the environmental influences on screen time and to determine whether interventions can be more successful at reducing screen time among young people if they also take into account relevant area-level factors.

The findings from this study suggest intervening upon high social and physical neighborhood disorder may be one relevant area-level factor to consider for future interventions. However, social and physical neighborhood disorder is a multifaceted issue that has many causes and consequences; therefore, the reduction of social and physical neighborhood disorder will require coordinated efforts from community members, law enforcement, and various other government departments [20]. One example of a coordinated effort between community members and law enforcement aimed at decreasing crime in some communities in Canada is the Neighbourhood Watch program [56]. This program is designed to strengthen community ties by having neighbors look out for other neighbors [56]. Implementing this program or similar programs along with other initiatives to lower fences and increase street lighting in neighbourhoods with high disorder may be one potential intervention strategy [30].

Along with efforts to decrease neighbourhood disorder, providing safe alternative opportunities to indoor screen time activities for youth who live in neighbourhoods with high disorder should also be considered. The after-school period has been identified as a key window of time for targeting reduction in screen time activities [57]. Therefore, the implementation of affordable community and/or school-based supervised after-school programs in neighbourhoods with high disorder may be another potential intervention strategy. While many youth in Canada (~80%) do not have access to a supervised after-school program [58], providing programs in areas with high neighbourhood disorder may be especially important for screen time reduction.

The multi-level analyses, the comprehensive measure of neighborhood disorder, the use of a large population-based sample, and the confirmatory sensitivity analyses are strengths of this study. A limitation of this study is the cross-sectional design, which limits the ability to make causal inferences about the relationships observed. Also, the use of self-report data for the screen time measures may have resulted in information bias. Similarly, some inaccuracies with the GIS data may have resulted in information bias of the physical neighbourhood disorder exposure variable. Any biases associated with these measures were likely non-differential, which would have led to the under-estimation of true associations [41]. Furthermore, other potentially important dimensions of neighborhood disorder were not included such as prostitution. Finally, the final sample was no longer representative of the population in terms of age, gender, ethnicity, SES, and urban-rural location.

## Conclusion

High social and physical neighborhood disorder predicted screen time use among a large population of youth. Therefore, high neighborhood disorder is one environmental factor that may be important to consider for future public health interventions and strategies aiming to reduce screen time among youth.

## Competing interests

The authors declare that they have no competing interests.

## Authors’ contributions

VC assisted with the design of the study, led the statistical analysis, and wrote the initial draft of the manuscript. IJ assisted with the design of the study, provided insight and guidance on the statistical analysis, and revised the manuscript for important intellectual content. Both authors approve the version that has been submitted.
